# Tolerability of SGLT2 inhibitors in patients with Fabry disease: An observational study

**DOI:** 10.1016/j.ahjo.2026.100795

**Published:** 2026-05-09

**Authors:** Isabel Mattig, Berit Zirkelbach, Carina Bonnekoh, Karl Stangl, Gerhard Hindricks, Fabian Knebel, Sima Canaan-Kühl, Sebastian Spethmann

**Affiliations:** a Deutsches Herzzentrum der Charité, Department of Cardiology, Angiology and Intensive Care Medicine, Charitéplatz 1, 10117, Berlin, Germany; b Charité – Universitätsmedizin Berlin, corporate member of Freie Universität Berlin and Humboldt-Universität zu Berlin, Charitéplatz 1, 10117, Berlin, Germany; c DZHK (German Centre for Cardiovascular Research), partner site Berlin, Germany; d Berlin Institute of Health at Charité – Universitätsmedizin Berlin, BIH Biomedical Innovation Academy, Berlin, Germany; e Sana Klinikum Lichtenberg, Innere Medizin II: Schwerpunkt Kardiologie, Berlin, Germany; f Charité – Universitätsmedizin Berlin, corporate member of Freie Universität Berlin and Humboldt-Universität zu Berlin, Medizinische Klinik mit Schwerpunkt Nephrologie und Internistische Intensivmedizin, Fabry Zentrum, Zentrum für seltene Nierenerkrankungen (CeRKiD), Campus Charité Mitte, Berlin, Germany

**Keywords:** Fabry disease, SGLT2 inhibitors, Heart failure

## Abstract

Cardiac involvement of Fabry disease (FD) includes heart failure (HF) treated according to general guideline recommendations. The retrospective study investigates HF medication in FD focusing on sodium glucose cotransporter 2 (SGLT2) inhibitors. HF medication, laboratory and echocardiographic measurements as well as side effects of SGLT2 inhibitors were analyzed at baseline and the last available follow-up. The analysis included 99 FD patients treated with angiotensin converting enzyme (ACE) inhibitors or angiotensin II type 1 receptor blocker (ARB) (50%), beta-blocker (31%), SGLT2 inhibitors (20%), diuretics (12%), angiotensin receptor-neprilysin inhibitors (ARNI) (2%), and mineralocorticoid receptor antagonists (MRA) (1%). After 663 (457–725) days, 15 FD patients treated with SGLT2 inhibitors showed stable cardiac and renal biomarkers. Echocardiographic parameters did not reveal a consistent pattern: Left ventricular ejection fraction (LVEF) showed a slight decrease in the SGLT2 inhibitor group and a significant reduction in patients without SGLT2 inhibitors. Left atrial volume index (LAVI) and tricuspid annular plane systolic excursion (TAPSE) showed an upward trend in the SGLT2 inhibitor group, whereas LAVI declined and TAPSE remained unchanged in patients without SGLT2 inhibitors. After propensity score matching, there were no inter- or intragroup differences. The most common side effects comprised polyuria, hypovolemia, and vertigo. Overall, these exploratory findings suggest an acceptable safety profile of SGLT2 inhibitors in this FD cohort, without allowing clear conclusions regarding clinical benefit.

## Highlights

• Sodium-glucose cotransporter-2 (SGLT2) inhibitors in Fabry Disease (FD) has been only sparsely investigated.
• This pilot study suggests that SGLT2 inhibitors are safe in patients with FD.
• However, no definitive conclusions can yet be drawn regarding their clinical benefit.

## 1 Background

Fabry disease (FD) is a rare X-linked lysosomal storage disorder characterized by reduced or absent α-galactosidase A activity [1]. The disease leads to the accumulation of globotriaosylceramide (Gb3) in multiple organs, including renal and cardiac involvement [1]. FD cardiomyopathy comprises left ventricular hypertrophy (LVH), heart failure with preserved ejection fraction (HFpEF) with progression into heart failure with mildly (HFmrEF) or reduced ejection fraction (HFrEF), and conduction disturbances [1]. Heart failure (HF) treatment is currently based on guideline recommendations for the general HF population and carries a higher risk of side effects, e.g., hyperkalemia and bradycardia, in FD patients [1]. New treatment options such as sodium glucose cotransporter 2 (SGLT2) inhibitors could prevent a progression in both renal and cardiac involvement [2]. Solomon et al. observed a reduction in decompensated HF and cardiovascular mortality with SGLT2 inhibitors, regardless of the HF phenotype and renal function [3]. Therefore, SGLT2 inhibitors now have a class I indication for HF treatment and represent the only recommended therapy for HFpEF – the leading phenotype in FD patients [1], [2]. However, data on HF medication, especially SGLT2 inhibitors, to treat patients with HF based on FD is rare. The present study aimed to evaluate HF medication in FD patients with a focus on tolerability and clinical response to SGLT2 inhibitors.

## 2 Methods

The current retrospective analysis included genetically confirmed FD patients with and without cardiac involvement, which was defined as left ventricular wall thickening (diastolic interventricular septum [IVSd] ≥12 mm). The primary endpoint comprised the proportion of patients with FD receiving SGLT2 inhibitor therapy. HF medication was assessed at baseline, the initial presentation after approval of SGLT2 inhibitors as HF therapy. Laboratory and echocardiographic measurements from clinical routine were collected at baseline and at the last available follow-up. Side effects while on SGLT2 inhibitor therapy were additionally assessed prospectively using a patient questionnaire. The institutional review board of the Charité – Universitätsmedizin Berlin, Germany, approved the study (EA1/219/23). Only patients who completed the questionnaire on side effects provided written informed consent, as the other part of the study was retrospective. Statistical analysis was performed in R Statistical Software (version 4.1.2 R Foundation for Statistical Computing, Vienna, Austria). Categorical variables are listed in percentages and continuous variables uniform as median with interquartile ranges (IQR). The inter- and intragroup comparison was conducted using Chi squared test, *t*-test for equal and unequal variances, Wilcoxon-test and Mann-Whitney-*U* test. The propensity score matching (PSM) was performed for better comparison of FD patients with and without SGLT2 inhibitors using the following covariates: gender, age, estimated glomerular filtration rate (eGFR) and E/e´. The intergroup comparison was conducted only in paired patients. The intragroup comparison was performed for patients with complete follow-up. The intergroup comparison was conducted as follows: 1) all available data were compared in the overall cohort and 2) only a complete data set of matched pairs was analyzed after PSM. A *p*-value of <0.05 was considered statistically significant.

## 3 Results

**Fig. 1:**
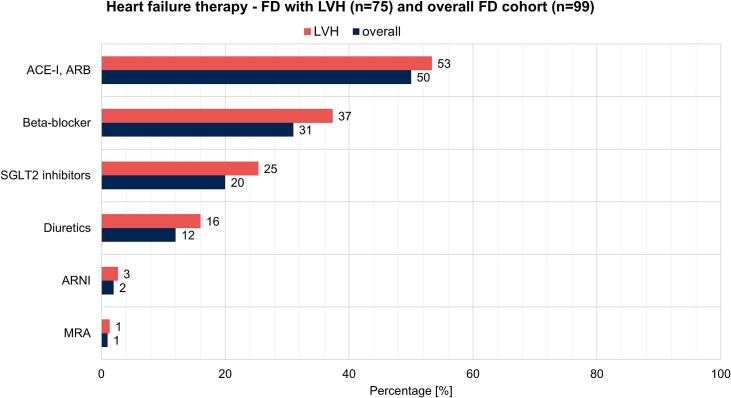
Distribution of heart failure therapy in the overall FD cohort and in the subgroup of FD patients with LVH. FD, Fabry disease; LVH, left ventricular wall thickness; ACE-I, angiotensin converting enzyme inhibitors; ARB, angiotensin II type 1 receptor blocker; SGLT2, sodium glucose cotransporter 2; ARNI, angiotensin receptor-neprilysin inhibitor; MRA, mineralocorticoid antagonist.

A total of 99 FD patients were enrolled in the current study. The median age of the overall cohort was 54 (42–64) years with 52% (*n* = 51) of patients being female. FD was classified as classic in 53% (*n* = 52), late onset in 28% (*n* = 28), and variant of unknown significance in 19% (*n* = 19) of patients. Overall, 36% of patients (*n* = 36) were in New York Heart Association (NYHA) class II or III, none of the patients reported NYHA class IV. Baseline characteristics are presented in Table 1. Heart failure therapy in the overall FD cohort and in the subgroup of 75 FD patients with LVH was as follows (Fig. 1): Angiotensin converting enzyme inhibitors (ACE-I) or angiotensin II type 1 receptor blocker (ARB) were prescribed in 50% (*n* = 49) of all FD patients and in 53% (*n* = 40) of LVH patients, beta-blocker in 31% (*n* = 31) and 37% (*n* = 28), SGLT2 inhibitors in 20% (*n* = 20) and 25% (*n* = 19), diuretics in 12% (*n* = 12) and 16% (*n* = 12), angiotensin receptor-neprilysin inhibitor (ARNI) in 2% (*n* = 2) and 3% (n = 2) as well as mineralocorticoid antagonist (MRA) in 1% (*n* = 1) and 1% (n = 1), respectively. Indications for SGLT2 inhibitors included HF (16%, n = 12), kidney disease (2%, n = 2), combined HF and kidney disease (1%, n = 1) as well as diabetes (1%, n = 1). SGLT2 inhibitors comprised dapagliflozin (85%, *n* = 17) and empagliflozin (15%, *n* = 3), both administered at 10 mg once daily.

**Table 1:** Baseline characteristics overall cohort.

	SGLT2 (−) (*n* = 79)	SGLT2 (+) (*n* = 20)	p-Value
Age [years]	52 (37–60)	64 (53–69)	0.001
Female, n [%]	44 (56)	7 (35)	0.103
BMI [kg/m^2^]	25 (22–29)	26 (24–31), n = 19	0.378
NYHA class	(*n* = 61)	(n = 17)	0.525
NYHA I, n [%]	40 (66)	10 (59)	
NYHA II, n [%]	16 (26)	4 (24)	
NYHA III, n [%]	5 (8)	3 (18)	
NYHA IV, n [%]	0 (0)	0 (0)	

*Comorbidities*			
Acute myocardial infarction, n [%]	3 (4)	1 (5)	0.816
Atrial fibrillation/flutter, n [%]	6 (8)	4 (20)	0.236
Bronchial asthma, n [%]	4 (5)	0 (0)	0.301
COPD, n [%]	6 (8)	4 (20)	0.105
Coronary heart disease, n [%]	8 (10)	3 (15)	0.549
Diabetes mellitus, n [%]	2 (3)	2 (10)	0.134
Dialysis, n [%]	1 (1)	0 (0)	0.608
Hypertension, n [%]	27 (35)	9 (45)	0.390
CIED, n [%]	6 (8)	3 (15)	0.313
PCI, n [%]	7 (9)	2 (10)	0.887
Peripheral artery disease, n [%]	1 (1)	0 (0)	0.611
Stroke, n [%]	15 (19)	4 (20)	0.938

*Fabry disease specific parameter*			
Acroparesthesia, n [%]	55 (76), *n* = 72	12 (63), n = 19	0.244
Angiokeratoma, n [%]	15 (25), *n* = 59	4 (21), n = 19	0.699
Cornea verticillata, n [%]	24 (42), *n* = 57	4 (21), n = 19	0.099
Gastrointestinal symptoms, n [%]	42 (58), *n* = 73	5 (25)	0.010
Hearing loss, n [%]	34 (52), *n* = 66	13 (65)	0.289
Hypohydrosis, n [%]	22 (32), *n* = 69	7 (35), n = 19	0.684
Pain crisis, n [%]	9 (14), *n* = 64	4 (20)	0.522

*Medication*			
ACE-I, ARB, n [%]	35 (44)	14 (70)	0.040
ARNI n [%]	1 (1)	1 (5)	0.289
MRA, n [%]	1 (1)	0 (0)	0.613
Beta-blocker, n [%]	20 (25)	11 (55)	0.011
Diuretics, n [%]	7 (9)	5 (25)	0.048
FD specific therapy, n [%]	42 (53)	11 (55)	0.883
SGLT2 medication, n [%]		Dapagliflozin 16 (80), Empagliflozin 4 (20)	
SGLT2 intake [d] (IQR)		285 (72–412)	


	SGLT2 (−) (n = 69) BL	SGLT2 (−) (*n* = 69) FUP	p-Value (intragroup comparison)	SGLT2 (+) (*n* = 15) BL	SGLT2 (+) (n = 15) FUP	p-Value (intragroup comparison)	p-Value BL vs. BL (intergroup comparison)	p-Value FUP vs. FUP (intergroup comparison)
*Baseline and follow*-*up overall cohort*								
NYHA class NYHA I, n [%] NYHA II, n [%] NYHA III, n [%] NYHA IV, n [%]	(n = 24) 13 (54) 8 (33) 3 (13) 0 (0)	(n = 24) 16 (67) 6 (25) 2 (8) 0 (0)	0.008	(n = 10) 6 (60) 3 (30) 1 (10) 0 (0)	(n = 10) 6 (60) 4 (40) 0 (0) 0 (0)	0.837	0.737	0.458

*Laboratory measurements*								
Follow-up period [d] (IQR)	952 d (630–1122)			308 d (184–579)				
Creatinine [mg/dL] (IQR)	0.9 (0.7–1.1), n = 67	0.9 (0.7–1.1), *n* = 67	0.704	1.0 (0.8–1.3), n = 13	1.1 (0.8–1.4), *n* = 13	0.724	0.801	0.390
NT-proBNP [pg/mL] (IQR)	79 (39–192), n = 51	76 (28–227), *n* = 51	0.800	810 (418–1362), n = 10	587 (527–1355), *n* = 10	0.575	0.483	0.022
Troponin T [pg/mL] (IQR)	7 (5–20), n = 45	8 (5–19), *n* = 45	0.494	42 (31–64), n = 11	44 (31–58), *n* = 11	0.767	0.046	0.005

*Echocardiography*								
Follow-up period [d] (±SD)	614 (455–881)			663 (457–725)				
LVEF [%] (IQR)	60 (56–65), n = 48	58 (54–62), *n* = 48	0.012	57 (54–60), n = 12	54 (47–60), *n* = 12	0.872	0.015	0.281
≥50%, n [%]	46 (96), n = 48	40 (83), *n* = 48	0.196	11 (92), n = 12	7 (58), n = 12	0.377	0.554	0.060
41–49%, n [%]	2 (4), n = 48	7 (15), *n* = 48	0.147	0 (0), n = 12	4 (33), *n* = 12		0.472	0.133
≤40%, n [%]	0 (0), n = 48	1 (2), *n* = 48		1 (8), n = 12	1 (8), *n* = 12		0.044	0.281
GLS avg [%] (IQR)	−16.0 (−21.0- −14.0), *n* = 9	−17.1 (−19.7- −12.9), *n* = 9	0.678	−12.4 (−15.6- -8.7), n = 9	−11.9 (−13.9- -8.6), n = 9	0.678	0.001	0.002
IVSd [mm] (IQR)	13 (10–15), *n* = 44	12 (10–15), *n* = 44	0.671	18 (16–21), *n* = 12	16 (15–22), *n* = 12	0.288	0.001	0.001
IVSd ≥ 12 mm, n [%] (IQR)	32 (73), n = 44	27 (61), n = 44	0.166	12 (100), n = 12	12 (100), n = 12	1.000	0.021	0.012
LAVI [mL/m^2^] (IQR)	36 (25–50), n = 42	32 (25–39), *n* = 42	0.046	33 (23–41), n = 8	41 (37–49), *n* = 8	0.027	0.106	0.007
TAPSE [mm] (IQR)	20 (18–24), n = 45	22 (17–25), *n* = 45	0.407	20 (18–22), *n* = 9	22 (22–24), n = 9	0.031	0.227	0.282

SGLT2, Sodium glucose cotransporter; NYHA class, New York Hear Association class; COPD¸ Chronic obstructive pulmonary disease; CIED, Cardiac implantable electronic device; PCI, Percutaneous coronary intervention; ACE-I, Angiotensin converting enzyme inhibitors; ARB, Angiotensin II type 1 receptor blocker; ARNI, Angiotensin receptor-neprilysin inhibitor; MRA, Mineralocorticoid receptor antagonists; FD, Fabry disease; IQR, Interquartile range; BL, Baseline; FUP, Follow-up; NT-proBNP, N-terminal pro-B-type natriuretic peptide; SD, standard deviation; LVEF, Left ventricular ejection fraction; GLS avg, Global longitudinal strain average; IVSd, Interventricular septum thickness at end-diastole; LAVI, Left atrial volume index; TAPSE, Tricuspid annular plane systolic excursion.

Baseline characteristics and follow-up measurements of patients with and without SGLT2 inhibitors are provided in Table 1. A follow-up examination (FUP) was performed in 15 FD patients with SGLT2 inhibitors and 69 FD patients without SGLT2 inhibitors. Exploratory analysis revealed no significant changes in laboratory parameters, including creatinine, NT-proBNP, and Troponin T in either group. Echocardiographic assessment suggested a significant decrease in left ventricular ejection fraction (LVEF) in patients without SGLT2 inhibitors, whereas LVEF showed a slight decrease in those receiving SGLT2 inhibitors. In the SGLT2 inhibitor group, left atrial volume index (LAVI) and tricuspid annular plane systolic excursion (TAPSE) increased significantly, while in patients without SGLT2 inhibitors, LAVI decreased and TAPSE remained largely unchanged (Table 1). After matching, 13 patients with and 13 without SGLT2 inhibitors were compared showing no significant changes in the inter- or intragroup comparison (Supplemental Table 1). Seventeen patients completed a questionnaire to evaluate side effects of SGLT2 inhibitors. Reported side effects, from most to least frequent, included polyuria (69%, *n* = 11), hypovolemia (47%, *n* = 7), vertigo (41%, n = 7), edema (38%, *n* = 6), hyperlipidemia (33%, *n* = 4), generalized pruritus (29%, *n* = 5), thirst (18%, *n* = 3), rash (13%, *n* = 2), syncope (12%, n = 2), urogenital infections (7%, n = 1), and constipation (6%,n = 1). Two patients (12%) discontinued the therapy due to fatigue and genital rash. None of the patients reported hypoglycemia or ketoacidosis.

## 4 Discussion

To conclude, this is the first study evaluating SGLT2 inhibitors in FD patients, the findings are largely descriptive and exploratory. SGLT2 inhibitors were prescribed due to HF, kidney disease, and diabetes. SGLT2 inhibitor discontinuation due to side effects was low (12%). The results are consistent with previous studies demonstrating good tolerability of the therapy [3]. Laboratory markers remained stable during follow-up in patients with and without SGLT2 inhibitors. Although LAVI and TAPSE increased under SGLT2 inhibitor therapy, LVEF showed a numerically slight decrease, suggesting the absence of a consistent trend among echocardiographic parameters. Positive results regarding a decrease in mortality and hospitalizations, as well as slight changes in NT-proBNP and eGFR were detected in other cardiomyopathies such as cardiac amyloidosis [4]. In FD, cardiomyopathy is divided into different disease stages, which include the accumulation of Gb3, followed by cardiac hypertrophy, an inflammatory response, and fibrosis [1]. SGLT2 inhibitors may reduce the disease progression as treatment effects comprise a reduction in cardiac inflammation, hypertrophy, and fibrosis [5].

Due to the small number of cases, the study is only hypothesis-generating. Further limitations comprise the retrospective study design with variable FUP periods. Side effects were mainly reported by patients which may lead to reporting bias. Therefore, further randomized controlled studies are mandatory to investigate the renal and cardiac effects of SGLT2 inhibitors in FD.

In summary, the present data suggests an acceptable safety profile of SGLT2 inhibitors in this FD cohort. However, no clear evidence of clinical benefit was observed, and the findings should therefore be interpreted as hypothesis-generating.

## CRediT authorship contribution statement

**Isabel Mattig:** Writing – review & editing, Writing – original draft, Supervision, Project administration, Methodology, Investigation, Formal analysis, Data curation, Conceptualization. **Berit Zirkelbach:** Writing – review & editing, Visualization, Methodology, Formal analysis, Data curation. **Carina Bonnekoh:** Writing – review & editing, Investigation, Data curation. **Karl Stangl:** Writing – review & editing, Supervision, Conceptualization. **Gerhard Hindricks:** Writing – review & editing, Supervision, Resources, Conceptualization. **Fabian Knebel:** Writing – review & editing, Supervision, Conceptualization. **Sima Canaan-Kühl:** Writing – review & editing, Investigation, Data curation, Conceptualization. **Sebastian Spethmann:** Writing – review & editing, Supervision, Project administration, Methodology, Investigation, Conceptualization.

## Ethical statement

The institutional review board of the Charité – Universitätsmedizin Berlin, Germany, approved the study (EA1/219/23). Only patients who completed the questionnaire on side effects provided written informed consent, as the other part of the study was retrospective.

## Funding

None.

## Declaration of competing interest

Isabel Mattig has received research grants from Pfizer Pharmaceuticals and Sanofi; and has received lecture fee and financial reimbursement for advisory board activities from Sanofi, Takeda, Pfizer Pharmaceuticals, BridgeBio, Chiesi Farmaceutici S.p.A, Amicus, and Bayer Vital GmbH outside the submitted work. Fabian Knebel received research funding from Pfizer Pharma GmbH and Sanofi, lecture fees from Amicus, Pfizer Pharma GmbH, AstraZeneca, Bayer Vital GmbH, Boehringer Ingelheim, Sanofi, Chiesi Farmaceutici S.p.A, Takeda, BMS, Canon, TomTec, Bracco, Novartis, and financial reimbursement for advisory board activities from Alnylam, BMS, and Pfizer Pharma GmbH, and for participation in clinical trials from AstraZeneca, SMT, Sanofi, Medtronic, Cardiac Dimensions, and LivaNova. Sebastian Spethmann received research funding from Pfizer Pharma GmbH, lecture fees from Novartis Pharma GmbH, AstraZeneca GmbH, Pfizer Pharma GmbH, Alnylam Netherlands B.V., Bayer Vital GmbH, travel fees from Pfizer Pharma GmbH, Alnylam Netherlands B.V., AstraZeneca GmbH and financial reimbursement for advisory board activities from Novartis Pharma GmbH, Pfizer Pharma GmbH, Bayer Vital GmbH.

## Appendix A Supplementary data

Supplemental Table 1 Baseline characteristics after PSM.
