# Supplementary material for: Tolerability of SGLT2 inhibitors in patients with Fabry disease: An observational study
Source: Am Heart J Plus. 2026 May 9;66:100795. doi: 10.1016/j.ahjo.2026.100795 (PMC13195756; doi:10.1016/j.ahjo.2026.100795)
Supplement: Supplemental Table 1 — Baseline characteristics after PSM. [file mmc1.docx]

| Supplemental Table 1: Baseline characteristics after PSM | | | | | | | | | | | |  |
| --- | --- | --- | --- | --- | --- | --- | --- | --- | --- | --- | --- | --- |
|  | | SGLT2 (-) (n=13) | | | SGLT2 (+) (n=13) | | | | p-value | | |  |
| *Age [Years]* | | 60 (49-66) | | | 60 (52-69) | | | | 0.350 | | |  |
| *Female, n [%]* | | 1 (8) | | | 2 (15) | | | | 0.546 | | |  |
| *BMI [kg/m²]* | | 24 (22-27) | | | 27 (24-32), n=12 | | | | 0.052 | | |  |
| *NYHA class*  *NYHA I, n [%]*  *NYHA II, n [%]*  *NYHA III, n [%]*  *NYHA IV, n [%]* | | (n=12)  3 (25)  5 (42)  4 (33)  0 (0) | | | (n=11)  7 (64)  3 (27)  1 (9)  0 (0) | | | | 0.145 | | |  |
| *Comorbidities* | | | | | | | | | | | |  |
| *Acute myocardial infarction, n [%]* | | 1 (8) | | | 0 (0) | | | | 0.308 | | |  |
| *Atrial fibrillation/ flutter, n [%]* | | 2 (15) | | | 2 (15) | | | | 1.000 | | |  |
| *Bronchial asthma, n [%]* | | 2 (15) | | | 0 (0) | | | | 0.141 | | |  |
| *COPD, n [%]* | | 2 (15) | | | 4 (31) | | | | 0.352 | | |  |
| *Coronary heart disease, n [%]* | | 3 (23) | | | 2 (15) | | | | 0.619 | | |  |
| *Diabetes mellitus, n [%]* | | 1 (8) | | | 2 (15) | | | | 0.539 | | |  |
| *Dialysis, n [%]* | | 1 (8) | | | 0 (0) | | | | 0.308 | | |  |
| *Hypertension, n [%]* | | 6 (46) | | | 7 (54) | | | | 0.695 | | |  |
| *CIED, n [%]* | | 2 (15) | | | 3 (23) | | | | 0.619 | | |  |
| *PCI, n [%]* | | 2 (15) | | | 1 (8) | | | | 0.539 | | |  |
| *Peripheral artery disease, n [%]* | | 0 (0) | | | 0 (0) | | | |  | | |  |
| *Stroke, n [%]* | | 3 (23) | | | 4 (31) | | | | 0.658 | | |  |
| *Fabry Disease specific parameter* | | | | | | | | | | | |  |
| *Acroparesthesia, n [%]* | | 9 (82), n=11 | | | 9 (70) | | | | 0.478 | | |  |
| *Angiokeratoma, n [%]* | | 3 (33), n=9 | | | 1 (8), n=12 | | | | 0.149 | | |  |
| *Cornea verticillata, n [%]* | | 2 (25), n=8 | | | 1 (8), n=12 | | | | 0.306 | | |  |
| *Gastrointestinal symptoms, n [%]* | | 7 (58), n=12 | | | 3 (23) | | | | 0.072 | | |  |
| *Hearing loss, n [%]* | | 8 (73), n=11 | | | 9 (70) | | | | 0.851 | | |  |
| *Hypohydrosis, n [%]* | | 6 (60), n=10 | | | 3 (25), n=12 | | | | 0.096 | | |  |
| *Pain crisis, n [%]* | | 4 (40), n=10 | | | 2 (15) | | | | 0.183 | | |  |
| *Medication* | | | | | | | | | | | |  |
| *ACE-I, ARB, n [%]* | | 6 (46) | | | 8 (62) | | | | 0.431 | | |  |
| *ARNI n [%]* | | 0 (0) | | | 1 (8) | | | | 0.308 | | |  |
| *MRA, n [%]* | | 0 (0) | | | 0 (0) | | | |  | | |  |
| *Beta-blocker, n [%]* | | 2 (15) | | | 8 (62) | | | | 0.016 | | |  |
| *Diuretics, n [%]* | | 2 (15) | | | 5 (39) | | | | 0.185 | | |  |
| *FD specific therapy, n [%]* | | 8 (62) | | | 7 (54) | | | | 0.691 | | |  |
| *SGLT2 medication, n [%]* | |  | | | Dapagliflozin 10 (77),  Empagliflozin 3 (23) | | | |  | | |  |
| *SGLT2 intake [d] (IQR)* | |  | | | 376 (237-502) | | | |  | | |  |
|  | |  | | |  | | | |  | | |  |
|  |  | | **Baseline and follow-up after PSM** | | | | |  | |  |  | |
|  | **SGLT2 (-) (n=13) BL** | | **SGLT2 (-) (n=13) FUP** | **p-value** | | **SGLT2 (+) (n=13) BL** | **SGLT2 (+) (n=13) FUP** | **p-value** | | **p-value BL vs. BL** | **p-value FUP vs. FUP** | |
| *NYHA class*  *NYHA I, n [%]*  *NYHA II, n [%]*  *NYHA III, n [%]*  *NYHA IV, n [%]* | (n=9)  3 (33)  3 (33)  3 (33)  0 (0) | | (n=9)  6 (67)  2 (22)  1 (11)  0 (0) | 0.136 | | (n=9)  6 (67)  2 (22)  1 (11)  0 (0) | (n=9)  6 (67)  3 (33)  0 (0)  0 (0) | 0.72 | | 0.055 | 0.711 | |
| *Laboratory measurements* | | | | | | | | | | | | |
| *Follow-up period [d] (IQR)* | *993 (644-1129)* | | |  | | *404 (210-658)* | |  | |  |  | |
| *Creatinine [mg/dL] (IQR)* | 1.0 (0.9-1.2), n=12 | | 0.9 (0.8-1.4), n=12 | 0.722 | | 1.2 (0.9-1.6) n=12 | 1.2 (0.9-1.7), n=12 | 0.346 | | 0.587 | 0.216 | |
| *NT-proBNP [pg/mL] (IQR)* | 184 (29-674), n=10 | | 199 (39-643), n=10 | 0.169 | | 810 (418-1362), n=10 | 587 (527-1355), n=10 | 0.374 | | 0.041 | 0.059 | |
| *Troponin T [pg/mL] (IQR)* | 26 (4-60), n=10 | | 28 (4-53), n=10 | 0.918 | | 40 (31-68), n=10 | 41 (31-60), n=10 | 1.000 | | 0.388 | 0.213 | |
| *Echocardiography* | | | | | | | | | | | | |
| *Follow-up period [d] (±SD)* | *645 (562-784)* | | |  | | *677 (500-731)* | |  | |  |  | |
| *LVEF [%] (IQR)* | 58 (51-64), n=11 | | 55.0 (49.0-58.0), n=11 | 0.247 | | 56 (53-60), n=11 | 53 (47-61), n=11 | 0.593 | | 0.788 | 0.363 | |
| *≥50%, n [%]* | 10 (91), n=11 | | 8 (73), n=11 | 0.521 | | 10 (91), n=11 | 6 (55), n=11 | 0.338 | | 1.000 | 0.095 | |
| *41-49%, n [%]* | 1 (9), n=11 | | 3 (27), n=11 | 0.521 | | 1 (9), n=11 | 4 (36), n=11 | 0.428 | |  | 0.293 | |
| *≤40%, n [%]* | 0 (0), n=11 | | 0 (0), n=11 |  | | 0 (0), n=11 | 1 (9), n=11 |  | |  |  | |
| *GLS avg [%] (IQR)* | -15.4 (-17.6-  -14.3), n=8 | | -14.5 (-17.8-  -12.0), n=8 | 0.161 | | -12.4 (-15.6-  -8.7), n=9 | -11.9 (-13.9-  -8.6), n=9 | 0.678 | | 0.046 | 0.108 | |
| *IVSd [mm] (IQR)* | 17 (13-21), n=10 | | 17 (13-19), n=10 | 0.625 | | 18 (17-21), n=11 | 16 (15-22), n=11 | 0.288 | | 0.703 | 0.450 | |
| *IVSd ≥ 12 mm, n [%] (IQR)* | 10 (100), n=10 | | 9 (90), n=10 | 1.000 | | 11 (100), n=11 | 11 (100), n=11 | 1.000 | | 1.000 | 0.283 | |
| *LAVI [mL/m²] (IQR)* | 44 (27-67), n=10 | | 32 (27-50), n=10 | 0.074 | | 45 (35-53), n=7 | 42 (37-50), n=7 | 0.553 | | 0.674 | 0.168 | |
| *TAPSE [mm] (IQR)* | 20 (17-25), n=11 | | 18 (17-26), n=11 | 0.811 | | 20 (21-23), n=8 | 22 (21-23), n=8 | 0.141 | | 0.613 | 0.151 | |

*PSM, Propensity score matching; SGLT2, Sodium glucose cotransporter; BMI, body mass index; NYHA class, New York Hear Association class; COPD¸ Chronic obstructive pulmonary disease; CIED, Cardiac implantable electronic device; PCI, Percutaneous coronary intervention; ACE-I, Angiotensin converting enzyme inhibitors; ARB, Angiotensin II type 1 receptor blocker; ARNI, Angiotensin receptor-neprilysin inhibitor; MRA, Mineralocorticoid receptor antagonists; FD, Fabry disease; IQR, Interquartile range; BL, Baseline; FUP, Follow-up; NT-proBNP, N-terminal pro-B-type natriuretic peptide; SD, standard deviation; LVEF, Left ventricular ejection fraction; GLS avg, Global longitudinal strain average; IVSd, Interventricular septum thickness at end-diastole; LAVI, Left atrial volume index; TAPSE, Tricuspid annular plane systolic excursion.*
